# Five‐year results of a modified left atrial maze IV procedure in the treatment of atrial fibrillation: a randomized study

**DOI:** 10.1111/ans.15486

**Published:** 2019-11-19

**Authors:** Dengshen Zhang, Jun Shi, Huayan Quan, Lu Liu, Jian Zhang, Yingqiang Guo

**Affiliations:** ^1^ Department of Cardiovascular Surgery West China Hospital, Sichuan University Chengdu Sichuan China; ^2^ West China School of Public Health and West China Fourth Hospital Sichuan University Chengdu Sichuan China

**Keywords:** atrial fibrillation, modified left atrial maze IV, surgical ablation

## Abstract

**Background:**

The left atrial maze IV (LAM‐IV) alone has been used to eliminate atrial fibrillation (AF) without severe right heart diseases. However, we felt that it could be improved and developed a modified LAM‐IV (MLAM‐IV). In this prospective trial, we aimed to investigate 5‐year clinical outcomes of AF in patients treated by the novel MLAM‐IV technique.

**Methods:**

Between September 2012 and October 2013, 120 patients who underwent valve surgery and bipolar radiofrequency ablation for AF were randomized into the LAM‐IV group (*n* = 60) or MLAM‐IV group (*n* = 60). At postoperative follow‐up examinations, data were recorded at 1, 3 and 6 months, and annually thereafter.

**Results:**

The mean ablation time and postoperative ventilation time were shorter in the MLAM‐IV group than in the LAM‐IV group (*P* < 0.001 and *P* = 0.03, respectively). At 5 years, the rate of freedom from AF was 69.0% in the MLAM‐IV group and 60.0% in the LAM‐IV group (hazard ratio 0.71, 95% confidence interval 0.39 to 1.32, *P* = 0.42). There were no differences with respect to the early operative mortality and major complications, late mortality, and major adverse events.

**Conclusions:**

The MLAM‐IV provides a technically simpler ablation process. The MLAM‐IV was associated with less ventilation support in the early postoperative period. The long‐term efficacy of the MLAM‐IV in the treatment of AF is comparable to that of the LAM‐IV.

## Graphical abstract

In this study, we aimed to investigate the outcomes of a modified left atrial maze IV procedure (MLAM‐IV) for the surgical treatment of AF. Results showed that the MLAM‐IV is technically simpler than the traditional left atrial maze‐IV (LAM‐IV) in the ablation process. The MLAM‐IV was associated with less ventilation support in early postoperative period. The long‐term efficacy of the MLAM‐IV in the treatment of AF is similar to that of the LAM‐IV.

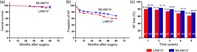

## Introduction

Atrial fibrillation (AF) is the most common cardiac arrhythmia. Patients with AF have twofold to fivefold increase risk for mortality.^1^ The maze procedure was introduced clinically by James Cox in the late 1980s to eliminate AF. After the initial procedure (maze‐I) and the second iteration procedure (maze‐II), the maze‐III achieves high freedom rate from AF.^2^ However, the maze‐III was not widely performed because of its complexity, which requires extensive cutting and sewing. To replicate the maze‐III lesion set using radiofrequency ablation, and to reduce technical difficulty, the maze‐IV is currently widely performed.^3^ Many surgeons have attempted to improve the maze‐IV to further simplify the procedure. Traditionally, the maze‐IV included biatrial (BA) ablation for AF.^3^ Studies have shown that only left atrial (LA) lesions have equal efficacy to BA lesions.^4^, ^5^, ^6^ Especially, in patients without severe right heart diseases, only the left atrial maze‐IV (LAM‐IV) is performed by some centres.

The LAM‐IV still has room for improvement in our clinical experience. From August to December of 2011, three patients were scheduled to undergo LAM‐IV, but their left pulmonary veins (PVs) could not be passed over and clamped with bipolar clamps because of their anatomic variation or adhesion to the LA posterior wall (LAPW). Consequently, the anterior wall of the PV and the LAPW were isolated as an entire box in those patients, replacing the left and right PVs separately, respectively. All patients were converted to sinus rhythm and were free from AF at 1 year.^7^ We called the new procedure as the modified left atrial maze IV procedure (MLAM‐IV). Given the easier ablation process in the modified procedure, this prospective trial was initiated in 2012 to evaluate the safety and long‐term clinical effectiveness of MLAM‐IV.

## Methods

### Trial design and patients

A prospective random trial was conducted on patients who had AF and were undergoing valve surgery between September 2012 and October 2013 in West China Hospital of Sichuan University, China. The trial was registered at the Chinese Clinical Trial Register (ChiCTR‐TRC‐12002742). It complied with the Declaration of Helsinki and was approved by the ethics committee of West China Hospital (Approval no. 2012183). Each patient provided written informed consent preoperatively. The trials design is shown in detail in Appendix S1.

### Surgical strategy

All patients underwent mitral and/or aortic replacement through median sternotomy by cardiopulmonary bypass with bicaval cannulation and a moderate hypothermia (32–34°C). After aortic cross‐clamping, the LA lesion set was performed under cardioplegic arrest. The lesion lines were ablated 4–6 times before the valve procedures. The LA appendage (LAA) and the ligament of Marshall were exposed and amputated routinely in both groups. The subsequent ablation of the lesion set was easier through the LAA incision. Patients with severe right heart diseases were excluded, and the RA lesion set was not created in this study.

**Figure 1:**
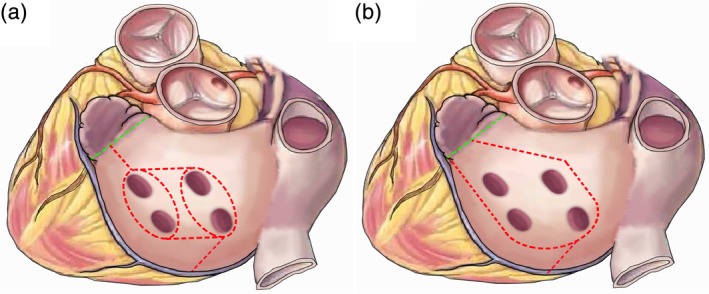
(a) The left atrial maze IV (LAM‐IV) procedure lesion set and (b) the modified LAM‐IV (MLAM‐IV) procedure lesion set.

**Figure 2:**
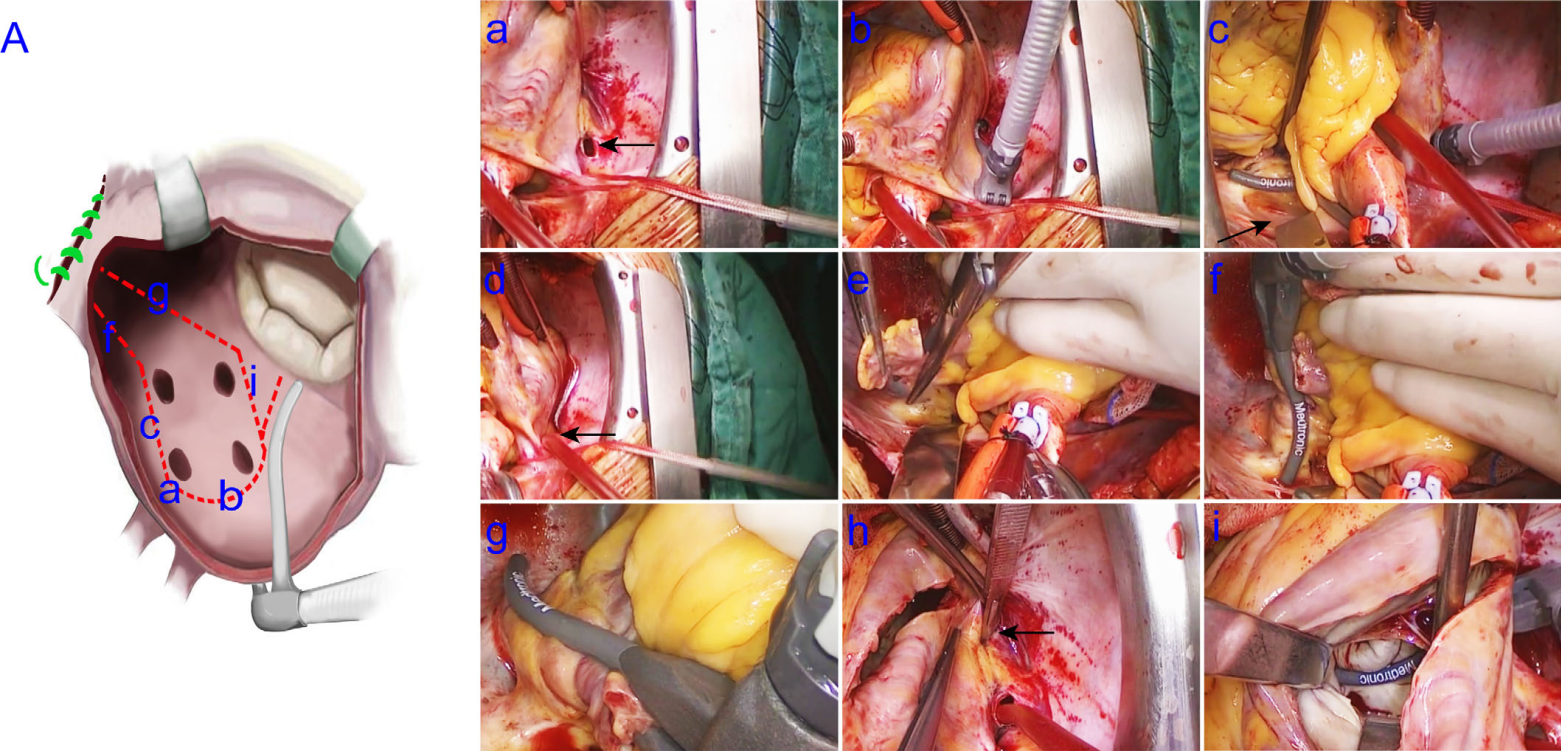
The MLAM‐IV procedure. Through a small incision in the superior right PV antrum (a, arrow), the anterior wall of the right‐sided PVs is ablated (b); the ligament of Marshall is dissected (c, arrow), and the LA roof line between the superior PVs is created (c). The incision in the superior right PV is also used as the LA aspirator (d, arrow). The LAA is amputated (e), connecting the lesion from the LAA to the LA roof line (f); a lesion is created on the anterior wall of the left‐sided PVs through the LAA incision (g). The LA floor lesion between the inferior PVs is created (i) through an incision (h, arrow), connecting the lesion from the right inferior PV to the mitral annulus. LA, left atrial; LAA, left atrial appendage; MLAM‐IV, the modified left atrial maze IV; PV, pulmonary vein.

The LAM‐IV lesion set was performed as previously described.^8^, ^9^ Briefly, as shown in Figure 1a, it included bilateral PV isolation after blunt dissection of the right and left PVs, surgical resection of the LAA, roof and floor lesions, and simultaneous ablation of a connecting lesion from the right lower PV to the mitral annulus. The MLAM‐IV lesion set was similar to the LAM‐IV, except that it did not require PV isolation alone, and each lesion was subjected to single‐layer ablation using a bipolar clamp. Detailed procedures for the MLAM‐IV lesion set (Fig. 1b) are described below (Fig. 2; Video S1).

### Postoperative care and follow‐up

Electrical cardioversion was performed immediately in patients who developed AF after the surgery and in those who remained to have AF at 7 days to 6 months. Patients attended scheduled outpatient visits at 1, 3 and 6 months and annually thereafter; follow‐up data were collected from outpatient clinic files. Freedom from AF based on the criterion no requirement for antiarrhythmic drugs was evaluated by 12‐lead ECG and by prolonged monitoring through 24‐h Holter ECG as recommended by consensus guidelines. Patients who displayed any atrial tachycardia lasting ≥30 s on Holter ECG were defined to have AF recurrence.^10^ The following data were recorded: questionnaire administration, 24‐h Holter ECG findings, 12‐lead ECG findings, thoracic echocardiography, early operative major complications within the initial 30 days after surgery (defined as death, excessive bleeding, reoperation, and low cardiac output), and late major adverse events (MAEs defined as death, excessive bleeding, reoperation, permanent stroke, permanent pacemaker implantation and heart failure).

### Statistics

Variables with continuous distributions were presented as means and standard deviation. Student's *t*‐test was used for continuous variables between the two groups. Pearson's Χ^2^ or Fisher's exact test was applied to compare the success rates between the two groups. The time‐to‐event analysis was performed using Cox proportional hazards regression to estimate hazard ratios and 95% confidence intervals (CIs). Kaplan–Meier curves were calculated to delineate cumulative rate of freedom from AF and incidence of death. All statistical tests were treated as two‐sided and were evaluated at a significance level of 0.05. Statistical analysis was performed using SPSS 22.0 (IBM, Chicago, IL, USA).

## Results

### Patient characteristics and perioperative results

A total of 120 patients were enrolled in the study and were randomized into two groups (MLAM‐IV group versus LAM‐IV group) between September 2012 and October 2013. Figure S1 shows the flow chart of patient enrolment. No significant differences were found in sex, age, New York Heart Association class, LA diameter or AF type between the two groups preoperatively (Table S1). The cardiopulmonary bypass time, operative time, and length of postoperative hospital stay were not significantly different between the two groups. The mean ablation time was shorter in the MLAM‐IV group than in the LAM‐IV group (18.5 ± 1.74 versus 16.6 ± 1.6 min, *P* < 0.001) (Table S2). The ventilation time was shorter in the MLAM‐IV group than in the LAM‐IV group (12.9 ± 3.4 versus 15.6 ± 5.5 h, *P* = 0.03) (Table S2). One patient in the LAM‐IV group and three patients in the MLAM‐IV group had anatomic variation of the PV and LAPW adhesion, respectively. However, the number of patients was small, making any valid comparison difficult.

### Early operative mortality and major complications

During the first 30 days, no deaths occurred in both groups. Major complications occurred in 1.7% (1 of 60) and 3.3% (2 of 60) in the MLAM‐IV and LAM‐IV groups, respectively (hazard ratio 2, 95% CI 0.18 to 22.06, *P* = 0.57). In the LAM‐IV group, one patient had low cardiac output and one patient underwent reoperation. In the MLAM‐IV group, one patient had excessive bleeding. All patients were discharged after treatment.

### Late mortality and major adverse events

**Figure 3:**
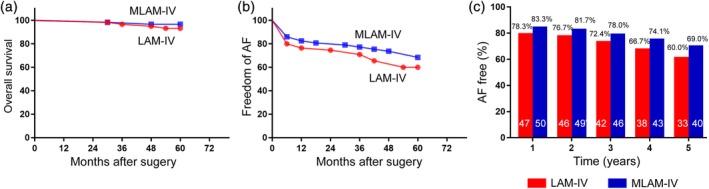
Freedom from AF recurrence in patients who underwent the MLAM‐IV (blue bars) versus the LAM‐IV (red bars). Kaplan–Meier overall survival curves for patients with LAM‐IV versus MLAM‐IV procedure (a). Kaplan–Meier AF free survival curves for patients with LAM‐IV versus MLAM‐IV procedure (b). The rate of freedom from AF group was slightly higher, but not significantly so, in the MLAM‐IV group from 1 to 5 years. The largest difference in freedom from AF was observed at 5‐year follow‐up (c). AF, atrial fibrillation; LAM‐IV, the left atrial maze IV; MLAM‐IV, the modified left atrial maze IV.

Five‐year follow‐up data were available for 99.2% (119 of 120) of the patients. During the 5‐year follow‐up, two deaths (3.3%) occurred in the MLAM‐IV group and four deaths (6.0%) in the LAM‐IV group (hazard ratio 0.5, 95% CI 0.09 to 2.71, *P* = 0.42). The two deaths in the MLAM‐IV group were due to heart failure and sepsis. For the LAM‐IV group, the causes of death were sepsis (50%), heart failure (25%) and respiratory failure (25%). The Kaplan–Meier curves of overall survival are shown in Figure 3a (hazard ratio 0.50, 95% CI 0.09 to 4.18, *P* = 0.42). The rate of cumulative MAEs over 5 years was 16.7% in the MLAM‐IV group and 23.3% in the LAM‐IV group (hazard ratio 0.68, 95% CI 0.30 to 1.52, *P* = 0.35). The most frequent causes of MAEs in the MLAM‐IV group were heart failure (30%), stroke (20%), bleeding (20%), sepsis (10%), reoperation (10%) and permanent pacemaker implantation (10%) and those in the LAM‐IV group were heart failure (28.6%), bleeding (21.4%), reoperation (21.4%), sepsis (14.2%), stroke (7.1%) and respiratory failure (7.1%).

### Freedom from AF at the primary efficacy endpoint

The overall freedom from AF for the entire cohort was 64.6% (73/113) at 5 years. The Kaplan–Meier curves of AF‐free survival are shown in Figure 3b (hazard ratio 0.72, 95% CI 0.39 to 1.32, *P* = 0.29). The rates of freedom from AF in the MLAM‐IV group versus LAM‐IV group were 83.3% (50/60) versus 78.3% (47/60) at 1 year (*P* = 0.49), 81.7% (49/60) versus 76.7% (46/60) at 2 years (*P* = 0.50), 78.0% (46/59) versus 72.4% (42/58) at 3 years (*P* = 0.50), 74.1% (42/58) versus 66.7% (38/57) at 4 years (*P* = 0.38) and 69.0% (40/58) versus 60.0% (33/55) at 5 years (*P* = 0.32), respectively. The differences were not statistically significant between the two groups. The largest difference in the rate of freedom from AF was observed at 5‐year follow‐up (9.0% higher in the MLAM‐IV group) (Fig. 3c). Thirty‐one and 38 patients received echocardiograms at 5 years in LAM‐IV group and in MLAM‐IV group, respectively. There were no significant differences in the left ventricular ejection fraction between the two groups (Fig. S2).

## Discussion

Despite the simpler procedure of the MLAM‐IV, the long‐term success rate of freedom from AF of the MLAM‐IV was not inferior to that of the LAM‐IV in our study and that of maze‐IV in other studies.^5^, ^6^, ^11^ The strong trend toward an increased duration of freedom from AF observed in the MLAM‐IV group may be attributed to the two main improvements. First, there was creation of four ablation lines to isolate all PVs and LAPW, which we considered as a novel ‘box lesion’. Second, each of the lesion set involves a single‐layer ablation, which could improve the transmurality of the lesions and reduce the gaps as possible.

The lesion set plays a key role in the surgical treatment of AF and prevents AF recurrence. A study indicated that 90% of the foci were detected in the PVs.^12^ Haïssaguerre *et al*. demonstrated that all atrial arrhythmias were attributed to the same focus firing irregularly and the majority of the foci were located in the atrium at the ostium of the PVs.^13^ However, studies showed that the 5‐year freedom from AF achieved by PV isolation alone was only around 55%.^14^, ^15^ PV isolation is the cornerstone of ablation technique, but it may not be sufficient. Many studies have confirmed that the LAPW plays an important role in the initiation of AF.^16^, ^17^ Embryologically, LAPW originates from the same cells of primordial PV,^18^ and spontaneous trigger activity and rotors from the LAPW have been reported in previous studies.^19^, ^20^ Many studies have shown that isolation of the LAPW leads to better results than isolation of only the PVs.^21^, ^22^ In our entire cohort, the MLAM‐IV resulted in a strong trend toward a longer duration of freedom from AF. We believed that isolation of the entire LAPW including the PVs is critical in preventing recurrence, which is superior to PV isolation alone. On the contrary, the lesion sets of MLAM‐IV, foci and reentry circuits in the LAPW and PVs are isolated from the cardiac electrophysiological system,^12^, ^13^, ^23^ which is crucial to prevent AF recurrence.

In our experience, MLAM‐IV avoids complete dissection of the PVs, and the PVs and atrial junctions, which may reduce the risk of PV rupture due to blunt dissection, particularly in patients who have anatomic variations of the PV or LAPW adhesions. Interestingly, the MLAM‐IV was associated with shorter ablation time and ventilation support in early postoperative period. The surrounding tissues of PVs were dissected completely and PVs were clamped, which aggravated the lung damage. The longer the ablation time, the more serious were the myocardial damage and pulmonary hypoxia, which could influence cardiac and lung function. The above reasons possibly led to the increased ventilation support in the LAM‐IV group.

The transmurality and continuity of the lesions play another crucial role in preventing AF recurrence. The success of surgical ablation depends on full‐thickness lesions leaving no conductive tissue gaps. The incomplete transmurality and continuity of the lesions were an important factor that leads to AF recurrence.^24^ In the LAM‐IV procedure, a bipolar clamp is placed around the PVs, and a double‐layer ablation is done in the LA antrum. The transmurality of the lesions is affected by the thickness of the myocardium; that is, the thicker the myocardium, the more difficult it is to achieve transmural lesions.^25^ Besides, by double‐layer ablation, the LAPW creases easily, which influences transmurality and continuity of the lesions. In the MLAM‐IV, each of the lesion set involves a single‐layer ablation using a bipolar clamp, which reduces the thickness of the ablated tissues and improves the transmurality and continuity of the lesions.

The LAA is cut routinely during ablation in our institution. The lesions from the LAA to the LA roof line and to the anterior wall of the left PVs were all ablated through the LAA incision, which makes the MLAM‐IV technically easier. The LAA is the source of more than 90% of intracardiac thrombus, particularly in AF. In a study of 987 patients who underwent repeat catheter ablation for AF, the LAA of 2.3% of the patients was the only source of AF.^26^ LAA was removed, which eliminated not only the most common source of thromboembolism, but also improved the success rate of freedom from AF.

In our centre, ablation of RA lesions is performed only in patients with severe right heart diseases, such as severe tricuspid regurgitation. Such patients were excluded from this study. In 10 studies included in a meta‐analysis, clinical outcomes of 2225 patients were available for comparative assessment. BA ablation appeared to be more effective than LA ablation in terms of freedom from AF at 1 year. However, the advantage of BA ablation was not observed beyond 1 year, and LA ablation significantly reduced the incidence of postoperative complications.^4^ The reason for the above‐mentioned result may be due to the fact that there was no RA enlargement in those cohort studies. In both groups in our study, the overall freedom from AF was 64.6% at 5 years, indicating that right‐sided lesions may not always necessarily occur in patients with mainly left heart disease.

## Limitations

Several limitations of the study require consideration. First, although the study was a prospective randomized trial, all procedures were performed in a single institution, which may create bias. Second, 24‐h Holter monitoring may be less effective in the detection of intermittent episodes of AF and asymptomatic arrhythmias. It carried a risk of overestimating success rates. Third, patients with severe tricuspid regurgitation and RA enlargement were excluded from this study, although it does not affect the comparison of clinical effects between the two operation methods, it may overestimate clinical efficacy of the left‐sided maze‐IV operation. Therefore, a multicenter large sample study for the modified operation increasing RA ablation with standard maze‐IV procedures as control is warrant to assess later results.

## Conclusions

The ablation process of MLAM‐IV is technically simpler than that of LAM‐IV. The MLAM‐IV was associated with less ventilation support in the early postoperative period. The long‐term efficacy of the MLAM‐IV in the treatment of AF is comparable to that of the LAM‐IV.

## Conflicts of interest

None declared.

## Supporting information


**Appendix S1.** The detailed trials design.

**Table S1.** Patients characteristics.

**Table S2.** Perioperative results between the left atrial maze IV (LAM‐IV) and modified LAM‐IV (MLAM‐IV) group.

**Figure S1.** Patient flow chart.

**Figure S2.** There were no differences in the preoperative left ventricular ejection fraction (LVEF) and 5‐years LVEF after surgery between the two groups.

**Video S1.** The modified LAM‐IV (MLAM‐IV) ablation procedure.
