# Supplementary material for: Five‐year results of a modified left atrial maze IV procedure in the treatment of atrial fibrillation: a randomized study
Source: ANZ J Surg. 2019 Nov 19;90(4):602–7. doi: 10.1111/ans.15486 (PMC7217219; doi:10.1111/ans.15486)
Supplement: Supplementary file 1 — Appendix S1. The detailed trials design. [file ANS-90-602-s001.docx]

**Zhang et al. Five-year results of a modified left atrial maze IV procedure in the treatment of atrial fibrillation: a randomized study**

**Supplementary trial design and patients**

A prospective random trial was conducted on patients who had AF and were undergoing valve surgery between September 2012 and October 2013 in West China Hospital of Sichuan University, China. The trial was registered at the Chinese Clinical Trial Register (ChiCTR-TRC-12002742). It complied with the Declaration of Helsinki and was approved by the ethics committee of West China Hospital (Approval no. 2012183). Each patient provided written informed consent preoperatively. For this study, all procedures were performed by Dr. Ying-qiang Guo. Ablation was performed using irrigated bipolar radiofrequency clamps (Cardioblate BP2, Medtronic, USA).

The exclusion criteria for participation were as follows: age<18 years, LA diameter >60 mm, severe tricuspid regurgitation, right atrial enlargement, history of cardiac surgery, additional coronary bypass surgery. Patients were randomly assigned to either the LAM-IV group or MLAM-IV group by a completely random method.^1^ The random sequence (numbers 1 to 120) was generated according to a random computer number generator, the first 60 random sequence were assigned to the LAM-IV group and last 60 random sequences to the MLAM-IV group, patients were numbered sequentially according to their admission time, they are then assigned to corresponding group. Amiodarone (200 mg/day, orally) was given postoperatively for 3 months with corrected QT interval monitoring.^2^ Warfarin was administered orally to maintain the international normalized ratio at 1.5–2.5. Freedom from AF based on the criterion no requirement for antiarrhythmic drugs at 1, 2, 3, 4, and 5 years was evaluated by 12-lead ECG and by prolonged monitoring through 24-h Holter ECG as recommended by consensus guidelines. Patients who displayed any atrial tachycardia lasting ≥30 s on Holter ECG were defined to have AF recurrence.^3^

Patients attended scheduled outpatient visits at 1, 3, and 6 months and 12 months and annually thereafter; follow-up data were collected from outpatient clinic files. The following data were recorded: questionnaire administration, 24-h Holter ECG findings, 12-lead ECG findings, annual thoracic echocardiography, early operative major complications within the initial 30 days after surgery, and late major adverse events (MAEs). Early operative major complications defined as death, excessive bleeding, reoperation, and low cardiac output. MAEs defined as death, excessive bleeding, reoperation, permanent stroke, permanent pacemaker implantation, and heart failure.

**Supplementary Reference**

1. Shuster. Design and analysis of experiments. *Methods Mol Biol.* 2007;**404**: 235-59.
2. Prystowsky EN, Padanilam BJ, Fogel RI. Treatment of Atrial Fibrillation. *JAMA.*2015;**314**:278-88.
3. Calkins H, Kuck KH, Cappato R, et al. 2012 HRS/EHRA/ECAS expert consensus statement on catheter and surgical ablation of atrial fibrillation: recommendations for patient selection, procedural techniques, patient management and follow-up, definitions, endpoints, and research trial design: a report of the Heart Rhythm Society (HRS) Task Force on Catheter and Surgical Ablation of Atrial Fibrillation. *Heart Rhythm.* 2012; **9**:632-696. e21.
