# Supplementary material for: Five‐year results of a modified left atrial maze IV procedure in the treatment of atrial fibrillation: a randomized study
Source: ANZ J Surg. 2019 Nov 19;90(4):602–7. doi: 10.1111/ans.15486 (PMC7217219; doi:10.1111/ans.15486)
Supplement: Supplementary file 2 — Table S1. Patients characteristics. [file ANS-90-602-s004.doc]

**Table S1 Patients characteristics**

|  | Group LAM-IV  （n=60） | Group MLAM-IV（n=60） | *P*-value |
| --- | --- | --- | --- |
| Sex(male/female) | 14/46 | 18/42 | 0.96 |
| Age(years) | 52.2±9.1 | 52.3±8.7 | 0.56 |
| LAD(mm) | 51.1±6.2 | 53.9±7.2 | 0.12 |
| LVEF（%） | 50.9±9.5 | 51.1±9.1 | 0.45 |
| AF duration(months) | 37.5±14.6 | 39.1±15.5 | 0.56 |
| Type of AF |  |  |  |
| Paroxysmal AF | 6 | 6 | 1.00 |
| Persistent AF | 19 | 23 | 0.44 |
| Long-standing persistent AF | 35 | 31 | 0.46 |
| Disease aetiology |  |  | 0.81 |
| RHD | 58 | 56 |  |
| Non­RHD | 2 | 4 |  |
| Disease aetiology |  |  | 0.81 |
| MVR+maze | 44 | 44 |  |
| AVR+maze | 2 | 4 |  |
| DVR+maze | 14 | 12 |  |

LAD: left atrial diameter; LVEF: left ventricular ejection fraction; AF: atrial fibrillation; RHD: rheumatic heart disease; MVR: mitral valve replacement; AVR: aortic valve replacement; DVR: mitral and aortic valve replacement.
