# Supplementary material for: Five‐year results of a modified left atrial maze IV procedure in the treatment of atrial fibrillation: a randomized study
Source: ANZ J Surg. 2019 Nov 19;90(4):602–7. doi: 10.1111/ans.15486 (PMC7217219; doi:10.1111/ans.15486)
Supplement: Supplementary file 3 — Table S2. Perioperative results between the left atrial maze IV (LAM‐IV) and modified LAM‐IV (MLAM‐IV) group. [file ANS-90-602-s005.doc]

**Table S2** **Perioperative results between the LAM-IV and MLAM-IV group**

RFT: radiofrequency ablation operation; CCT: aortic across-clamp time; CPB: cardiopulmonary bypass.

|  | Group LAM-IV  （n=60） | Group MLAM-IV（n=60） | *P*-value |
| --- | --- | --- | --- |
| RFT（min） | 18.5±1.7 | 16.6±1.6 | <0.001 |
| CCT（min） | 80.0±23.7 | 79.3±30.0 | 0.67 |
| CPB time（min） | 115.1±31.6 | 109.9±31.7 | 0.53 |
| Ventilation time（h） | 12.9±3.4 | 15.6±5.5 | 0.03 |
| ICU stay（day） | 2.0±0.7 | 1.8±0.7 | 0.69 |
| Hospital stay time(day) | 9.7±5.9 | 9.9±6.0 | 0.90 |
