# Supplementary figures and images for: Five‐year results of a modified left atrial maze IV procedure in the treatment of atrial fibrillation: a randomized study
Source: ANZ J Surg. 2019 Nov 19;90(4):602–7. doi: 10.1111/ans.15486 (PMC7217219; doi:10.1111/ans.15486)

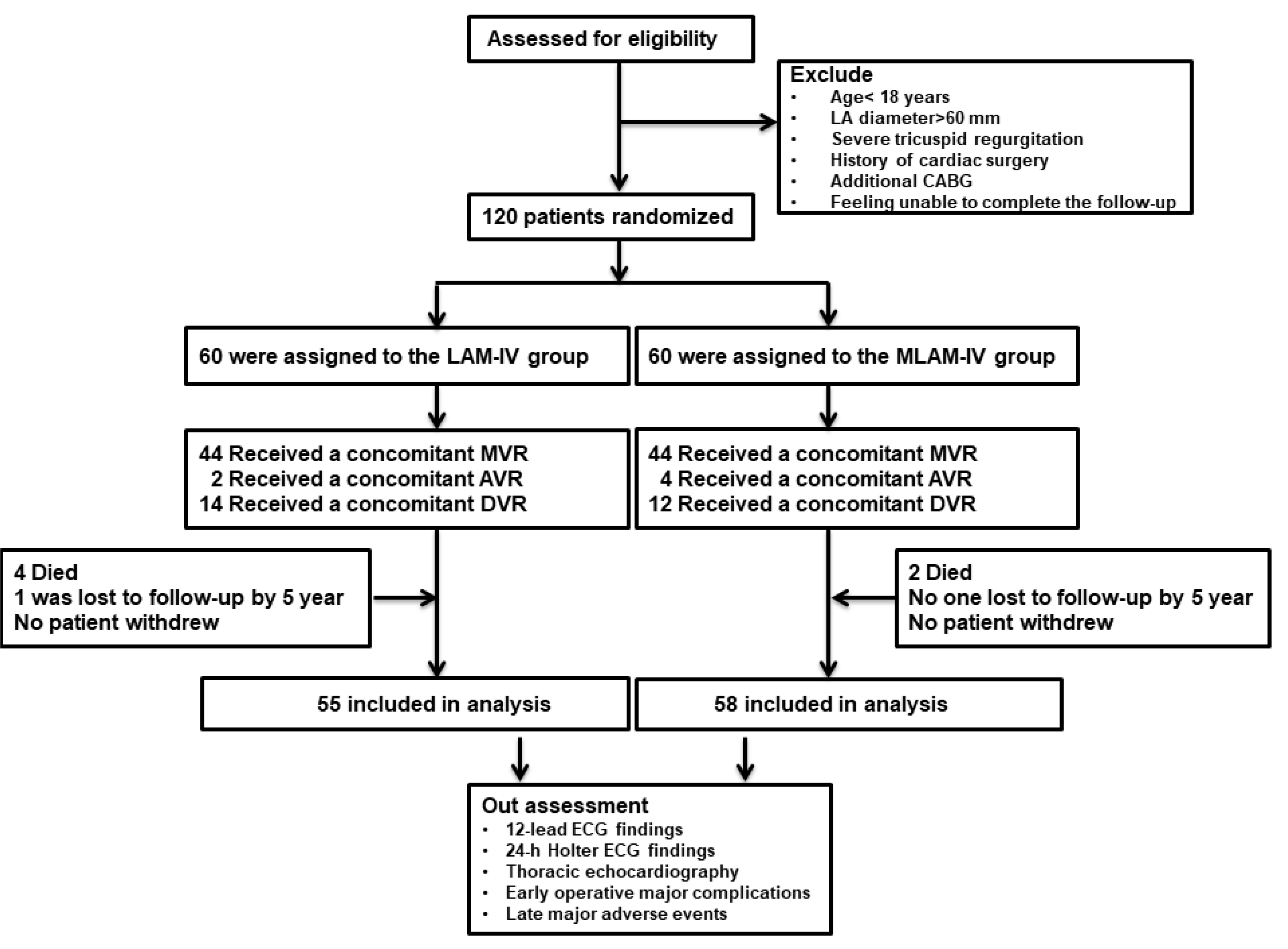

Supplement: Supplementary file 4 — Figure S1. Patient flow chart. [file ANS-90-602-s002.tif]

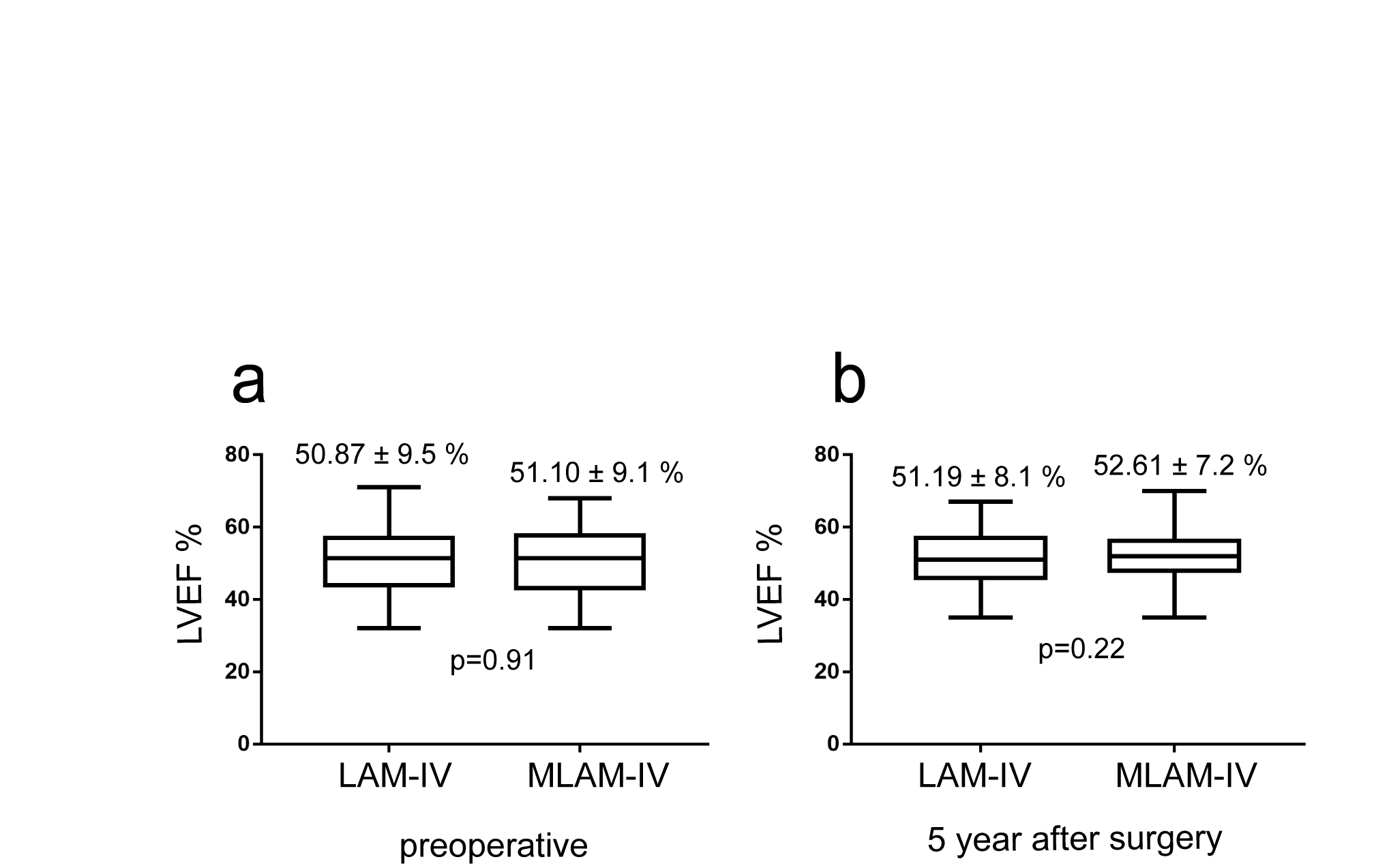

Supplement: Supplementary file 5 — Figure S2. There were no differences in the preoperative left ventricular ejection fraction (LVEF) and 5‐years LVEF after surgery between the two groups. [file ANS-90-602-s003.tif]
